# Preclinical Pharmacokinetic Considerations for the Development of Antibody Drug Conjugates

**DOI:** 10.1007/s11095-014-1584-z

**Published:** 2014-12-02

**Authors:** Amrita V. Kamath, Suhasini Iyer

**Affiliations:** Department of Preclinical and Translational Pharmacokinetics and Pharmacodynamics, Genentech, Inc, 1 DNA Way (Mailstop 463A), South San Francisco, CA 94080 USA

**Keywords:** antibody drug conjugate, biotherapeutics, cancer, pharmacokinetics, preclinical

## Abstract

Antibody drug conjugates (ADCs) are an emerging new class of targeted therapeutics for cancer that use antibodies to deliver cytotoxic drugs to cancer cells. There are two FDA approved ADCs on the market and over 30 ADCs in the clinical pipeline against a number of different cancer types. The structure of an ADC is very complex with multiple components and considerable efforts are ongoing to determine the attributes necessary for clinical success. Understanding the pharmacokinetics of an ADC and how it impacts efficacy and toxicity is a critical part of optimizing ADC design and delivery i.e., dose and schedule. This review discusses the pharmacokinetic considerations for an ADC and tools and strategies that can be used to evaluate molecules at the preclinical stage.

## Introduction

**Fig. 1:**
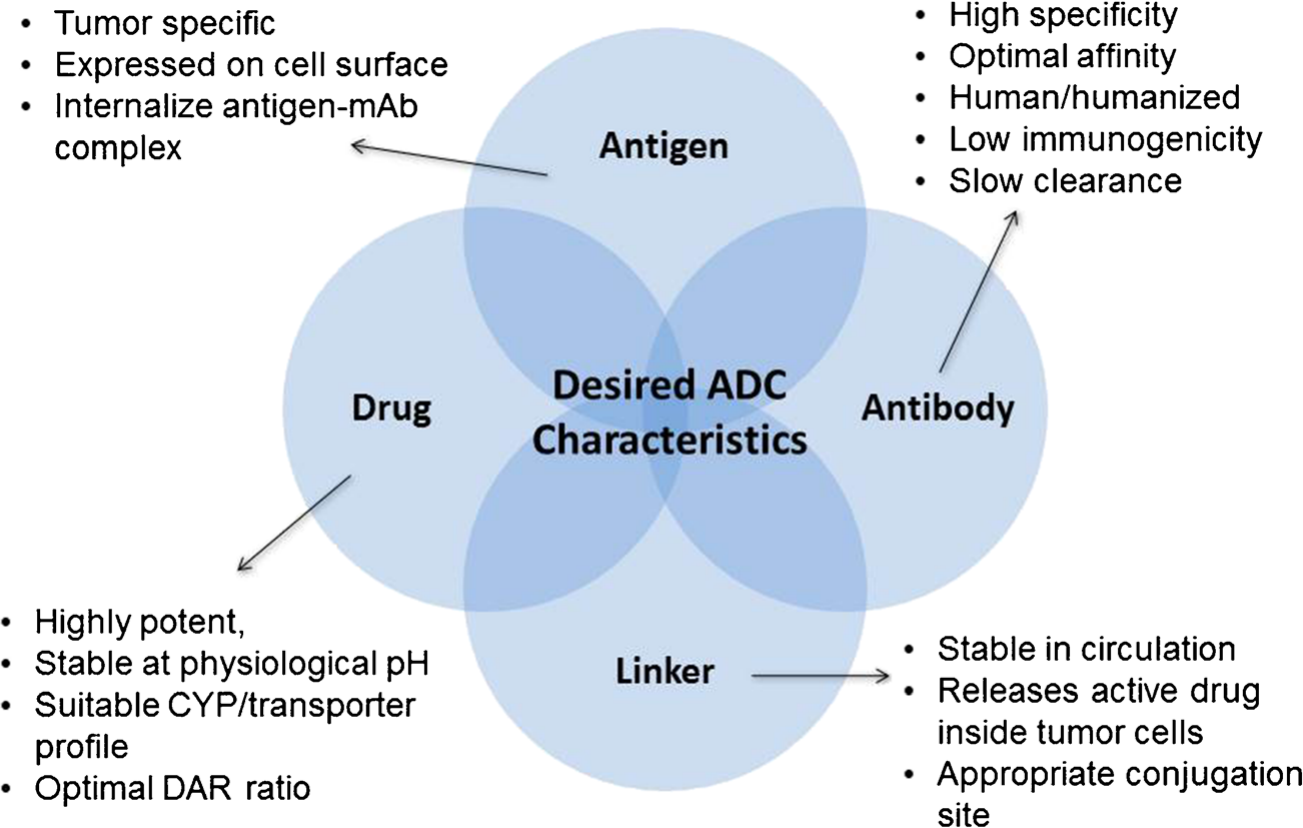
Desired attributes of the components of an ADC.

Antibody drug conjugates (ADCs) are a promising class of antibody related therapeutics for cancer that combine the antigen targeting specificity and favorable pharmacokinetic properties of monoclonal antibodies with the cytotoxic potential of small molecule chemotherapeutics (1–3). The vision of ADCs is to provide targeted delivery of the cytotoxic agent to tumor tissue and spare normal tissue, thereby decreasing its toxicity and improving its therapeutic window. The design of an ADC is critical in delivering on this vision and there is a lot of research focused on the optimal design of the molecule and its main components i.e., the antibody directed to an antigenic target, the cytotoxic drug and the linker that attaches the antibody to the drug (4–6). Some considerations for each component (antibody, linker, drug) as well as the molecule as a whole are highlighted in Fig. 1. An important question in the development of ADCs is to define the exposure-response relationship for both efficacy and safety. Understanding the pharmacokinetics of the ADC, exposure at the site of action and drivers of efficacy and toxicity are important to address this key question, to further enable the design of a better molecule. Additionally, this can be used for optimizing dose and regimen to help realize the promise of an ADC therapeutic.

Mylotarg® (gemtuzumab ozogamicin) was the first ADC to be approved in 2000 for the treatment of acute myeloid leukemia (AML) and was composed of a CD33-targeted antibody linked to the cytotoxic drug calicheamicin via an acid-labile hydrazone linker. It was later withdrawn from the market in 2010 over concerns of safety and failure to reproduce its clinical benefit. There are currently two FDA approved ADCs on the market, Adcetris® (brentuximab vedotin) approved in 2011 for the treatment of Hodgkin’s Lymphoma and anaplastic large-cell lymphoma, and Kadcyla® (ado-trastuzumab emtansine) approved in 2013 for the treatment of HER+ metastatic breast cancer. Adcetris® is a CD30-targeted antibody linked to an auristatin (monomethyl auristatin E, MMAE) via a protease cleavable linker, and Kadcyla® is a HER2-targeted antibody (trastuzumab) linked to a maytansinoid derivative (DM1) via a non-cleavable thioether linker.

The clinical pipeline has more than 30 ADCs at various stages of development from Phase 1 to Phase 3 and many more ADCs at the preclinical stage (7,8). The field is rapidly evolving and tremendous effort is being put into applying insights from more advanced ADCs to guide the design of next generation ADCs. Some of the modifications being explored include novel cytotoxins, linkers, different sites of conjugation, and antibodies to novel antigenic targets. Several design features of an ADC impact its pharmacokinetics that could then impact its efficacy and toxicity (5,9). One important example is the choice of linker which ideally should be stable in circulation, but release the active drug inside the tumor cell. The types of linkers being explored are cleavable or non-cleavable, with varying degrees of stability. The site of conjugation on the antibody also has an impact on stability of the linker with different sites conferring varying degrees of stability to the ADC.

In this review we discuss the pharmacokinetic considerations in the development of ADCs and the strategies and tools that can be employed to evaluate them at the preclinical stage. We also briefly discuss the bioanalytical considerations and commonly used methods for pharmacokinetic assays.

## Bioanalytical Considerations

In addition to being complex molecules, ADCs are also heterogeneous mixtures comprising of multiple species with varying numbers of drugs per antibody (drug to antibody ratio, DAR) as well as different sites of drug linkage arising from different conjugation chemistry approaches such as conjugation through lysines (Kadcyla®) or cysteines derived from reduced internal disulfide bonds (Adcetris®), or site specific conjugation (10). These heterogeneous and dynamic characteristics of an ADC result in a unique set of bioanalytical challenges requiring multiple bioanalytical assays. In order to adequately characterize the pharmacokinetics of an ADC, and answer the key question on exposure-response relationships, it is critical to understand what analytes are relevant, what needs to be measured, and at what stage of development. The bionalytical strategies for the development of ADCs have been the subject of intense discussion and are highlighted in several recent papers including a comprehensive review by Kaur and colleagues at Genentech and a white paper by the ADC working group of the American Association of Pharmaceutical Scientists (11–13).

**Fig. 2:**
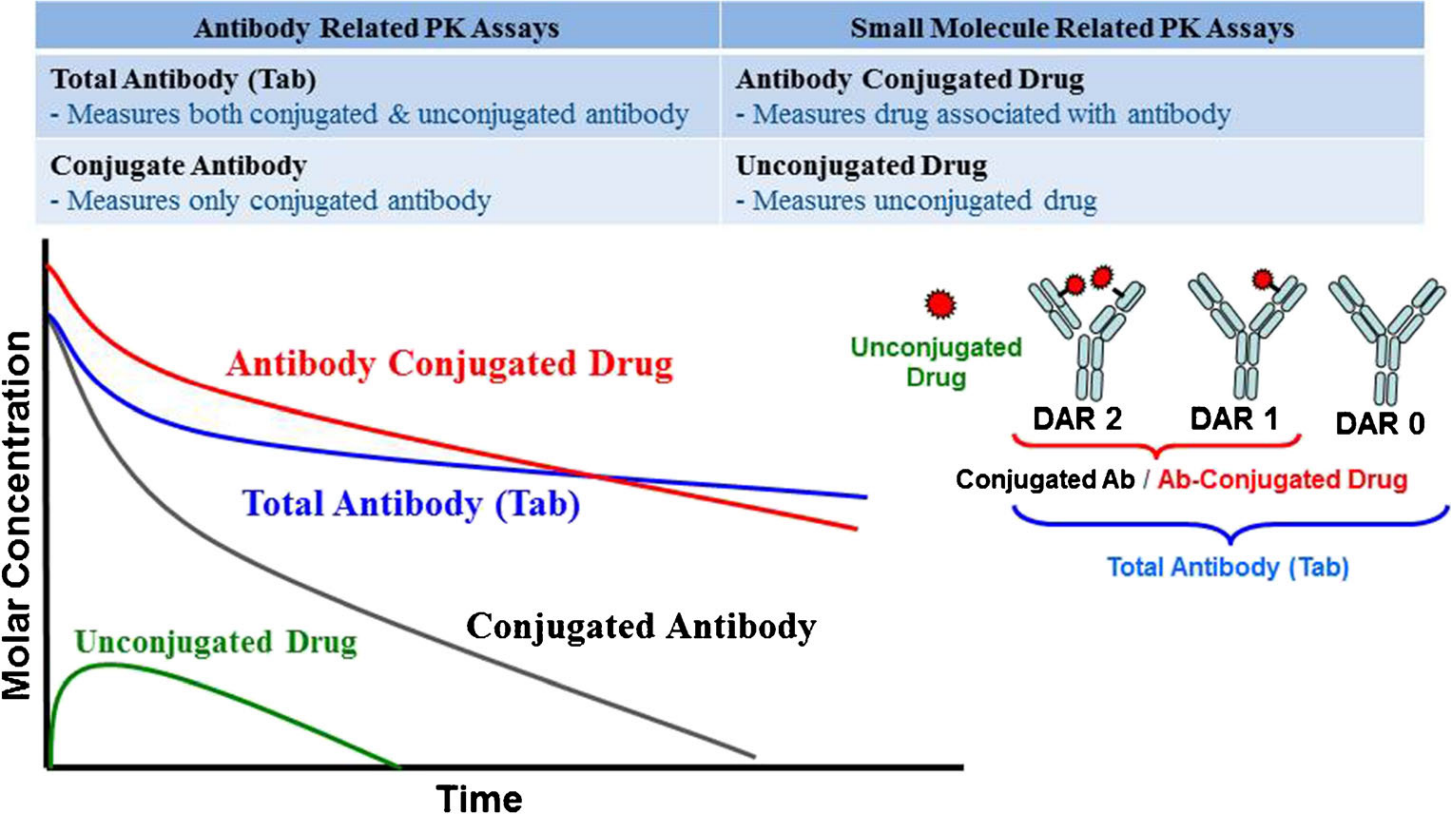
Pharmacokinetic profiles of different analytes.

The analytes commonly used for evaluation of ADC PK and their associated PK profiles are shown in Fig. 2 (9,11). They include antibody related analytes such as i) total antibody (Tab) which measures both conjugated and unconjugated antibody and ii) conjugated antibody which measures antibody that has at least one drug attached to it (i.e., ≥DAR1), and small molecule related analytes such as i) antibody conjugated drug which measures any drug associated with the antibody and ii) unconjugated drug. The analytical methods used to measure these analytes include ELISA as well as LC/MS/MS methods.

Additional complexity is introduced by *in vivo* biotransformation of an ADC due to deconjugation and catabolism. This results in changing of the initial DAR distribution caused by loss of one or more drugs, as well as formation of several new species such as catabolites and metabolites, linkers and linker drugs, adducts with endogenous molecules such as albumin, cysteine, and complexes with any soluble/shed target antigen and other antibodies (11). Since the DAR distribution can continue to change *in vivo*, with possible impact to the clearance, distribution, and activity of the ADC, it is important to develop appropriate methods to measure this. Novel bioanalytical assays have been developed to characterize DAR distribution such as affinity capture capillary LC-MS and affinity capture hydrophobic interaction chromatography (HIC) (11,14–16). In addition to DAR distribution, these assays can also provide insights into the mechanisms of drug loss e.g., maleimide exchange and formation of adducts with albumin or cysteine for ADCs using maleimide chemistry (11,17).

At the preclinical stage, when the ADC design is still being optimized, it is valuable to use all these assays to characterize the pharmacokinetics as well as gain a mechanistic understanding of deconjugation and catabolism processes. With further development and availability of more information on the mechanisms of biotransformation, exposure-response relationships, as well as the correlation between various analytes, the types of bioanalytical assays employed could be determined on a case-by case basis depending on the type of information required at that stage (13).

## Pharmacokinetic Properties of ADCs

**Fig. 3:**
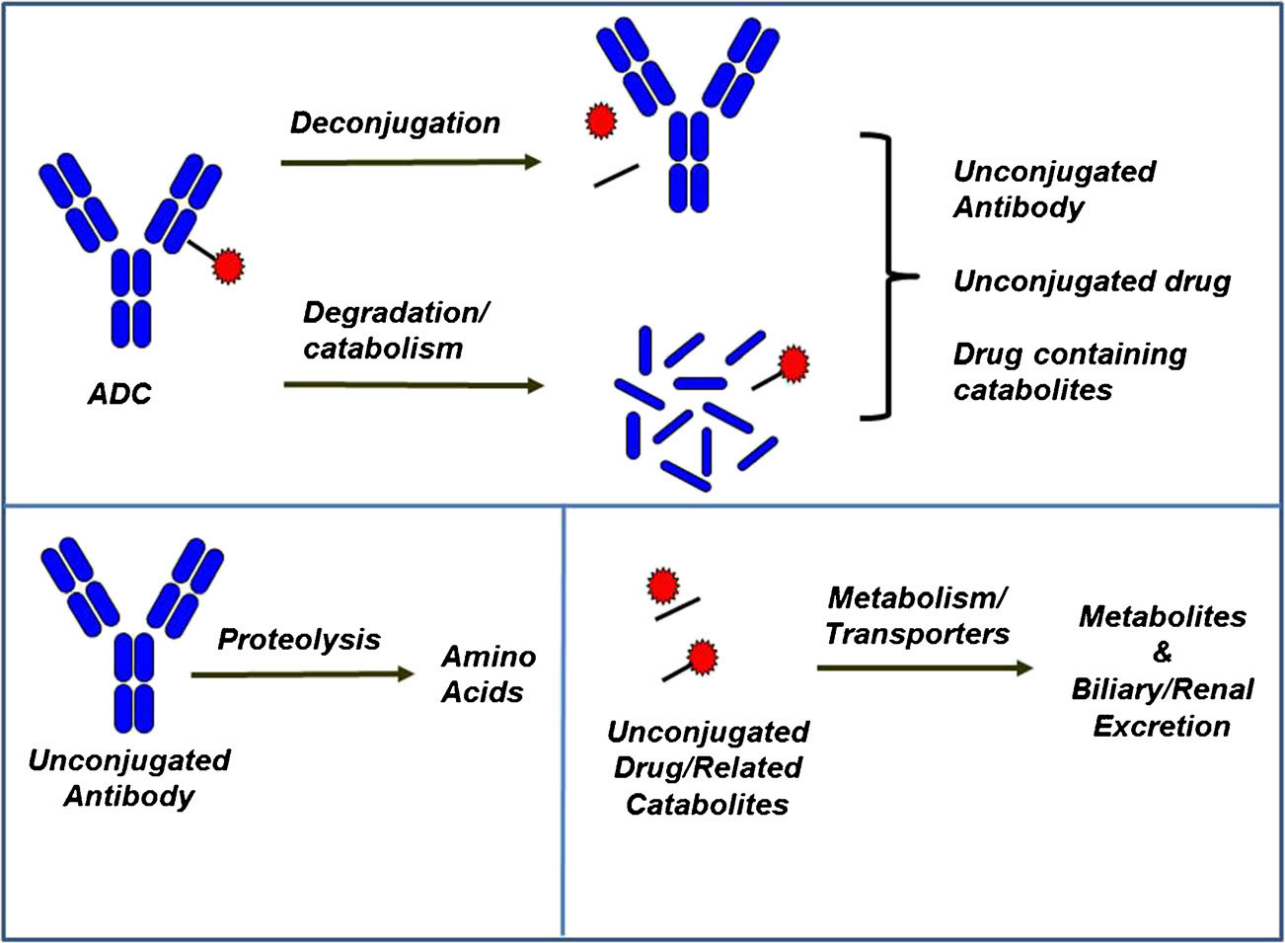
Proposed disposition of an ADC.

The primary mechanism of action of an ADC is binding to its specific antigen target on tumor cells and internalization via receptor mediated endocytosis followed by trafficking from endosomes to the lysosomes where the cytoxic drug is released into the cell causing cell death (1). The ADC however can also be taken up into cells (with or without target expression) non-specifically via pinocytosis, which could lead to unwanted drug release in non-target cells. Such non-specific uptake and release of potent drug could contribute to toxicity. The theoretical ADC elimination pathways based on nonclinical and clinical data are shown in Fig. 3 and include deconjugation and degradation or catabolism through nonspecific or target-mediated proteolysis (9). These processes could take place to varying extents in circulation and/or intracellularly depending on the characteristics of the ADC components (5,17,18). Deconjugation of the ADC leads to the formation of unconjugated antibody and unconjugated drug, while catabolism of the ADC leads to the formation of antibody fragments or drug containing catabolites. The unconjugated antibody or antibody fragments can further undergo proteolysis to generate/release amino acids. The cytotoxic drug and drug related catabolites can undergo metabolism via CYP or non-CYP enzymes or be transported by transporters like P-gycoprotein and get excreted via the biliary or renal route.

The overall PK characteristics of an ADC such as slow clearance, long half-life and limited tissue distribution are driven by its antibody component. In addition to the antibody component, there are additional elimination mechanisms related to its linker and drug component. The characteristics of an ADC that can influence its pharmacokinetics and factors that should be considered during its preclinical development are shown in Table I and are discussed in more detail below. The PK parameters with the conjugated antibody analyte of Adcetris® and Kadcyla® across multiple species are shown in Table II.

**Table I:** Influence of ADC Characteristics on its Pharmacokinetics

ADC characteristic	PK considerations	Assessment
Antibody	PK of naked antibody should be consistent with expected IgG PK (non-target mediated disposition)	Characterize PK-dose dependency: target affinity, target expression/turnover, no off-target binding
Linker	i) Conjugation can impact the PK of an antibody depending on the type of conjugation chemistry utilized. ii) Linker should have suitable stability to deliver ADC to target to minimize toxicity but have sufficient lability to release active drug once internalized	i) Assess impact of conjugation of the PK of the naked antibody ii) Assess linker stability *in vitro* and *in vivo* iii) Evaluate what is being released (active drug, catabolites) iv) Evaluate mechanism for instability across species
Site of conjugation	Conjugation at some sites stabilizes the linker possibly due to differences in solvent accessibility and local charge.	Assess linker stability *in vitro* and *in vivo*.
Drug load (DAR)	Higher DAR species associated with faster clearance and increased toxicity	Assess *in vivo* pharmacokinetics and tissue distribution
Cytotoxic drug	i) MOA of the drug can impact the PK driver of efficacy/toxicity e.g. tubulin binding agents *vs.* DNA damaging agents ii) Release of the active drug and any relevant metabolites could also impact PK drivers	i) Assess metabolites, DDI potential (CYP inhibition/ induction/ reaction phenotyping) ii) P-gp substrate/inhibitor (other transporters)

**Table II:** ADC Pharmacokinetic parameters of Kadcyla® and Adcetris® in Rat, Monkey, and Human

Characteristic	Kadcyla®	Adcetris®
Target	HER2	CD30
Antibody isotype	IgG1	IgG1
Cytotoxic drug	DM1	MMAE
Linker	MCC (thioether- noncleavable)	MC-vc-PAB (protease cleavable)
Avg DAR	3.5	4
Pharmacokinetic parameters (Analyte: conjugated antibody)		
Rat		
Dose CL (mL/day/kg) T1/2 (day) Vss (mL/kg)	20 mg/kg single IV dose 13–15 4.6 62–64	5 mg/kg single IV dose 19.8 8–15 –
Monkey		
Dose CL (mL/day/kg) T1/2 (day) Vss (mL/kg)	30 mg/kg IV q3W 9.4–10.5 4.6–5.2 68–70	3 mg/kg single IV dose 14.3–21.4 1.6–2.7 –
Human^a^		
Dose CL (mL/day/kg) T1/2 (day) Vss (mL/kg)	3.6 mg/kg IV q3W 12.9 ± 3.4 3.5 ± 0.8 60 ± 13.6	1.8 mg/kg IV q3w 25.1 4.43 117
References	(19,20)	(21,22)

^a^Human PK parameters from Phase 1 studies in Cycle 1 at doses at or near MTD

### Antibody

Characteristics related to antibody biology that affect the PK of an ADC include antibody structure, binding affinity and specificity to the antigen, FcRn binding, and Fcgamma interaction that drive effector functions (23,24). The relatively long life of an ADC compared to a small molecule is due to recycling via FcRn, which protects it from catabolism in the lysosomes. As with antibodies, the ADC can be taken up into cells specifically via receptor mediated endocytosis (target-dependent mechanism) or non-specifically via pinocyctosis (target-independent mechanism). FcRn-bound ADC can be recycled back to the cell surface and released back into circulation while ADC that is not bound to FcRn undergoes proteolytic degradation in the lysosome. Another similarity of ADCs to antibodies is the phenomenon of target mediated drug disposition where the interaction of the antibody with the target antigen impacts its pharmacokinetics including its clearance and distribution (25). This typically results in non-linear PK with higher clearance at lower doses and a decrease in the clearance at higher doses once the target is saturated. Also similar to an antibody, the tissue distribution of an ADC is limited with the initial distribution in the vascular space followed by slow diffusion across vascular endothelial cells into tissues. This can also be influenced by binding to and internalization by the target antigen (23,24). Other factors that impact PK and tissue distribution of an ADC that are similar to an antibody are i) presence of soluble and/or shed antigen in circulation that can form immune complexes on binding to the ADC (26,27), and ii) immunogenicity to the ADC (formation of anti-therapeutic antibodies, ATAs) that can increase its clearance and decrease exposure (24).

**Fig. 4:**
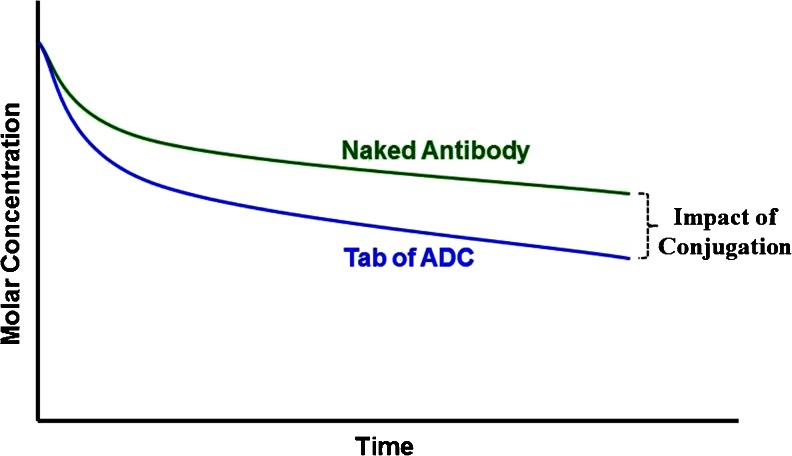
Effect of conjugation on the PK profile of an antibody.

The selection of the right antibody that has pharmacokinetic properties that are consistent with expected IgG behavior is very crucial to ensure optimal PK properties of the ADC. The pharmacokinetics of an antibody may be altered by the conjugation of the drug (28) and this impact can be discerned by comparing the PK profiles of the naked antibody and the Tab of the ADC as shown in Fig. 4. The further apart the two curves, the larger is the impact of conjugation on the antibody. Conjugation can also impact the tissue distribution as has been observed for some ADCs when compared to the naked antibody (29,30). Several factors that could contribute to this change including drug load, hydrophobicity among others have been investigated and are discussed in more detail below.

### Linker

**Fig. 5:**
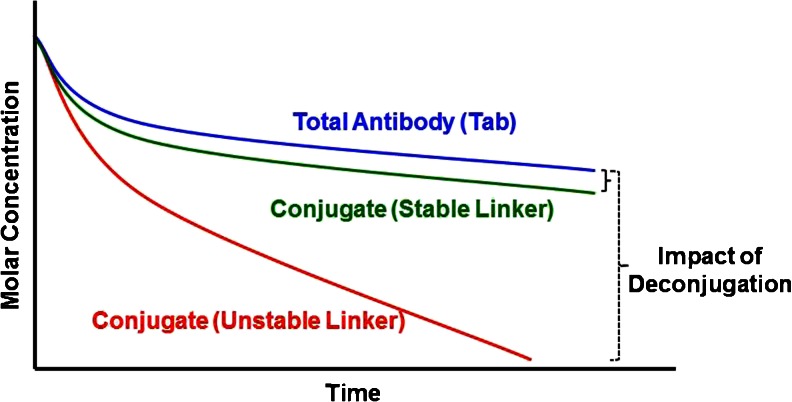
PK profiles of ADCs with different linker stability.

An important consideration impacting the pharmacokinetics of an ADC is the stability of linker. Ideally the linker should be stable in circulation to minimize toxicity but release the active cytotoxin once it is internalized by the tumor cell. The linkers can be categorized as cleavable and non-cleavable, with varying degrees of stability (1,31). Cleavable linkers use mechanisms within the cells or cellular compartments to release active cytotoxin, such as low pH (acid labile linkers), glutathione levels (disulfide linkers), and lysosomal proteases (protease cleavable linkers). In contrast, for non-cleavable linkers (e.g. thioether linkers), the entire ADC has to be degraded to release the active cytotoxin. The three advanced ADCs all use different linker types (1,31): peptide based protease cleavable linker (Adcetris®, linker: MC-vc-PAB), non-cleavable thioether linker (Kadcyla®, linker: MCC), and acid-labile hydrazone linker (Mylotarg®, linker: AcBut). The comparison of the Tab profile with the conjugated antibody profile allows for an assessment of linker stability as shown conceptually in Fig. 5, where the conjugated antibody concentrations for the more unstable linker decline much faster compared to that of the stable linker.

Different types of linkers used on the same antibody can impact its pharmacokinetics as illustrated by the following examples. The pharmacokinetics of anti-HER2 ADCs was determined in mice using disulfide linkers with different hindered structures (32). The clearance of the ADC decreased with the increase in the degree of hindrance of the disulfide linker as follows: SPDP-DM1 (least hindered) > SPP-DM1 > SSNPP-DM3 > SSNPP-DM4 (most hindered). The MCC-DM1 linker (nonreducible thioether) had similar pharmacokinetics in this study as the SSNPP-DM4 (most hindered disulfide). Similar results were seen in other studies where the pharmacokinetics of a disulfide linker (SPP) was compared to a thioether linker (MCC) linking the cytotoxic drug DM1 to two different antibodies, anti-CD22 and anti-HER2 in rats and mice, respectively (33,34). The Tab clearance for each of the antibody was similar regardless of the linker, however the ADC clearance (i.e., conjugated antibody clearance) was faster for SPP-DM1 compared to MCC-DM1, for both anti-CD22 and anti-HER2 ADCs as shown in Table III. Another interesting observation was different metabolite profiles for different linker types, with the cleavable linkers releasing cytotoxic drug, and the non-cleavable linkers releasing the drug attached to an amino acid: e.g., maytansinoid ADCs with the non-cleavable linker MCC-DM1 produced only lysine-MCC-DM1 whereas ADCs with cleavable linkers SPP-DM1 and SPDB-DM4 produced multiple metabolites including lysine-SPP-DM1, DM1, S-methyl-DM1, S-methyl-DM1-sulfoxide and S-methyl-DM1-sulfone (for mAb-SPP-DM1), and lysine-SPDB-DM4, DM4, S-methyl-DM4, S-methyl-DM4-sulfoxide and S-methyl-DM4-sulfone (for mAB-SPDB-DM4) (35). Some of these metabolites could also enhance anti-tumor activity via bystander killing mechanisms where the cytotoxic agent released in one cell diffuses to neighboring cells and exerts its effect (34,35).

**Table III:** Effect of Linker on PK of Tab and Conjugate in Mice (Anti-Her2 ADCs) and Rats (Anti-CD22 ADCs)

Molecule	Linker type	Tab CL mL/day/kg	Conjugate CL mL/day/kg
Anti-HER2 ADC PK in mouse^a^: dose of 3 mg/kg			
Trastuzumab-SPP-DM1	Cleavable (disulfide)	8.5	41
Trastuzumab-MCC-DM1 (T-DM1)	Non-cleavable	8.4	19
Anti-CD22 ADC PK in rat^a^: dose of 2000 μg/m^2^ of drug (approximately 20 mg/kg of ADC)			
CD22-SPP-DM1	Cleavable (disulfide)	13	67
CD22-MCC-DM1	Non-cleavable	11	18
CD22-MC-MMAF	Non-cleavable	11	29
CD22-MC-vc-PAB-MMAE	Cleavable (protease)	24	90

^a^References: (33,34)

As highlighted in these examples, during optimization of an ADC, the linker type needs to be carefully evaluated with regard to its stability, impact on catabolism, and how that affects its pharmacokinetics.

### Cytotoxic Drug

While the cytotoxic drug does not drive the PK of the ADC *per se*, its MOA can impact the PK drivers of efficacy and toxicity. The two main categories of cytotoxins currently being explored are microtubule inhibitors (auristatins, maytansinoids) and DNA damaging agents (calicheamicin, duocarmycins, anthracyclines, pyrrolobenzodiazepine dimers). Unlike microtubule inhibitors that preferentially kill proliferating cells, DNA damaging agents can also kill non-proliferating cells, potentially giving them a different spectrum of efficacy as well as toxicity. This could play a role in changing the PK driver of efficacy as well as toxicity and emphasizes the importance of investigating appropriate dosing regimens to optimize efficacy and minimize toxicity depending on the type of cytotoxin used. The three most advanced ADCs use different cytotoxins: calicheamicin (Mylotarg®), auristatin (MMAE, Adcetris®), and maytansinoid (DM1, Kadcyla®). Most of the ADCs in the clinical pipeline also use these three types of cytotoxins: calicheamicin, DM1, DM4, MMAE (7). SG-CD33A, a novel CD33 targeting ADC with a pyrolobenzodiapine dimer and using site specific conjugation with engineered cysteines has recently entered Phase 1 trials in AML patients and it will be interesting to see the pharmacokinetic and safety profiles of this ADC with a novel cytotoxin and linker (36,37). In addition to the MOA of these different cytotoxins, molecule characteristics such as permeability, metabolism, and whether it is a P-glycoprotein substrate could also play important roles in determining bystander effects and resistance development (1,31).

### Drug Load and Site-Specific Conjugation

The conventional processes to conjugate linker-drugs to antibodies (lysine conjugation or conjugation to cysteines derived from reduction of inert-chain disulfide bonds) produces ADCs with heterogeneous mixtures of multiple species with different drug loads: DAR of 0–8 (10).

Each of these DAR species could potentially have distinct PK properties and activities (28,38). While *in vitro* potency could increase with increase in drug load per antibody, it does not always follow that *in vivo* efficacy and safety profiles will also improve (28). Hambelet *et al.* (28), made ADCs with an anti-CD30 antibody (cAC10) conjugated to MMAE with the MC-vc-PAB linker with either two, four, or eight drugs per antibody. ADCs with lower drug loads (DAR of 2 or 4) had slower clearance values, longer half-lives and were better tolerated in mice compared to an ADC with a higher drug load (DAR of 8). In addition, the DAR4 ADC showed equivalent *in vivo* antitumor activity to the DAR8 ADC at equal antibody doses despite having half the amount of MMAE. Similar results were seen in a rat study with trastuzumab-MC-vc-PAB-MMAF conjugates (DAR of 2, 4, and 6), where the ADCS with the higher drug loads cleared faster and were less tolerated compared to conjugates with lower drug loads (39).

The next generation ADC efforts have focused on eliminating this heterogeneity by using site-specific conjugation methods to produce a more homogenous ADC to improve stability, PK, and therapeutic index, as shown in several recent studies (17,29,40,41). These conjugation strategies include the use of engineered cysteines, unnatural amino acids, and enzymatic conjugation through glucotransfersases and transglutaminases (10). A recent study at Genentech using site-specific conjugation with engineered cysteines (THIOMAB™ technology) showed that chemical and structure dynamic of the conjugation site can influence the stability of the ADC (17). Three thio-trastuzumab-MC-vc-MMAE THIOMABs were generated (DAR of 1.7–1.9) using engineered cysteines at three different sites (Fc-S396C, LC-V205C, HC-A114C), differing in solvent accessibility and local charge. The conjugate at a highly solvent accessible site (Fc-S396C) was the most unstable in plasma and allowed maleimide exchange of the linker drug with reactive thiols in albumin, free cysteine, or reduced glutathione. Other ADCs using maleimide chemistry such as anti-CD30-MC-MMAF and anti-CD70-MC-MMAF have also shown adduct formation in plasma, such as albumin-MC-MMAF and cys-MC-MMAF (42,43). The conjugate at a partially accessible site with a positively charged environment (LC-V205C) prevented this maleimide exchange by promoting succinimide ring hydrolysis and was the most stable in plasma. The stability of the third conjugate at a partially accessible site with a neutral environment (HC-A114C) was in between the other two and showed both mechanisms. The stability of these variants corresponded with their *in vivo* activity with the more stable conjugate showing greater *in vivo* efficacy in mouse xenograft models compared to the least stable conjugate. Other site-specific conjugation methods such as use of transglutaminase have also shown that conjugation site has an impact on ADC stability and pharmacokinetics (44).

Conjugation can also impact tissue distribution of the antibody and several studies have shown a trend towards slightly increased hepatic uptake of ADCs (29,30,42). This was seen with auristatin (29,42) and calicheamicin conjugates (30). In contrast, the maytansinoids conjugated to the antibody through lysine residues (e.g. Kadcyla®, SAR3419, IMGN901) showed tissue distribution profiles similar to the naked antibody (45,46).

For auristatin conjugates, tissue distribution studies have been conducted using the protease cleavable MC-vc-PAB-MMAE linker with different drug loads (DAR 3.1 *vs.* DAR 1.7) and different conjugation methods (reduced interchain disulfides *vs.* site specific conjugation through engineered cysteines) (29). Since increased hepatic uptake was seen with conjugates with lower drug loads as well as different conjugation methods compared to the naked antibody, one proposed hypothesis was that higher hydrophobicity of the drug conjugates compared to the naked antibody leads to a greater clearance by the reticuloendothelial system (29). A recent study by Seattle Genetics (47) to explore increased ADC clearance with higher drug loading seen for the MC-vc-auristatin linkers showed a correlation between hydrophobicity and plasma clearance. The rapid plasma clearance with higher drug loading (DAR of 8) seen with MC-vc-MMAF linker (more hydrophobic), was slightly less pronounced with MC-MMAF (slightly less hydrophobic) and was not seen with a novel auristatin T-based drug linker (AT-GLu-MDpr) which was designed to minimize hydrophobicity. In addition, the AT-Glu-MDpr linked conjugate with high drug load showed similar hepatic uptake as its parent antibody in a perfused liver system. They also showed that full reduction of the interchain disulfides of an antibody, without any drugs attached to it, did not increase clearance of the antibody. This study indicates that increased clearance and hepatic uptake could be attributed to the intrinsic hydrophobicity of the drug linker as hypothesized by several groups (29,31), and not due to destabilization of the antibody structure due to reduction of the interchain disulfides. Additional studies with different antibodies as well as *in vivo* tissue distribution studies are needed to confirm these interesting findings.

## Preclinical PK Strategy to Evaluate ADCs

At the preclinical stage, it is important to understand the pharmacokinetics of an ADC in conjunction with its *in vitro* and *in vivo* activity to gain insights into its mechanism of action and help optimize and select the right ADC. ADC PK is usually characterized in non-clinical species used for efficacy and safety studies. The choice of the species for these studies usually faces similar challenges as with the naked antibody in terms of appropriate antigen binding i.e., when some species may be a non-binding species. However, the non-antigen dependent process should be similar in binding or non-binding species. To adequately characterize the PK of an ADC, it is critical to have the appropriate tools. As discussed previously, there have been great advances in the types of analytical methods to measure the different components of the ADCs like the total antibody, conjugated and unconjugated drug, DAR distribution, catabolites and metabolites in various matrices such as plasma, bile, and tissues from *in vitro* or *in vivo* studies depending on the different stages of drug development (11–13). Additional work is still needed to better understand the best analytes to use for exposure-response correlations and to better understand *in vitro*-*in vivo* correlations as well as cross-species correlations.

Types of studies that can be done preclinically to characterize the PK of ADCs include the following:

i. *In vitro* stability studies in plasma from different species to understand linker stability as well as mechanisms of deconjugation across species.
ii. *In vitro* catabolism studies to determine the types of catabolites/metabolites formed and whether they have any activity in *in-vitro* cell potency assays.
iii. *In vivo* PK and exposure of the various analytes in the efficacy and toxicity species to characterize the PK, determine PK drivers of efficacy/toxicity, establish *in vitro*-*in vivo* correlations of stability and mechanisms of deconjugation/catabolism.
iv. Biodistribution studies to look for tumor and normal tissue uptake (specific or non-specific), and *in vivo* catabolite profiles in various tissues, including understanding any contribution of catabolites to any bystander effects.
v. *In vitro* potency, CYP, and transporter profiling of the cytotoxic drug to evaluate the risk of possible drug-drug interactions in the clinic.
vi. Utilize *in vivo* exposure data at the efficacious and toxic doses to estimate therapeutic index.
vii. Prediction of human PK to estimate efficacious dose and schedule in patients.

In addition to studies to characterize the PK of ADCs, it is also important to integrate PK, efficacy and toxicity data to answer the key question on exposure-response relationships and translation from preclinical species to patients. Model based approaches can be used to integrate this data and further our mechanistic understanding of the pharmacology. Depending on the stage of development, different questions become important (e.g., questions on target selection, antibody affinity, linker stability, E-R relationship) and the type of model employed will depend on the type of question that needs to be addressed. For example: early on in development, systems pharmacology models can be used to enable target selection, whereas classic PKPD models can be used later on to understand exposure-response relationships as well as translational PKPD to predict human PK as well as efficacious dose and dose regimens in patients. The modeling of ADC PK is complicated due to the its multiple elimination pathways including deconjugation and catabolism, as well as multiple analytes with their distinct PK properties (conjugated antibody with multiple DAR species, unconjugated drug, unconjugated antibody, *etc.*). Tremendous efforts are ongoing in developing PK/PD models to guide the development of ADCs. Some of the models that have been proposed include semi-mechanistic PK models using a series of transit compartments to describe the deconjugation process from higher to lower DAR species (48,49), simplified models with a one-step deconjugation process (48,50), target-mediated drug disposition models (51), and multi-scale mechanistic PK/PD models (52,53). A key limitation for the use of some of these models is the availability of appropriate data, or tools to obtain that information. However, as more clinical data becomes available and tools to obtain these types of data improve, sophisticated models can be developed and applied to expand our understanding of the key analytes that correlate with efficacy and safety.

## Conclusion

The ADC field is rapidly expanding with many molecules at various stages of development. With limited clinic data on a small number of ADCs, the guidelines and strategies for developing these molecules are still evolving. This review has highlighted several areas where PK of an ADC makes an impact on its activity. There have been huge strides in this field, especially with new linker technology, site-specific conjugation techniques to create more homogenous ADCs, as well as tremendous advances in novel bionalytical tools to measure PK of various analytes and methods to integrate the available PKPD information. Nevertheless, many questions still remain on PK drivers of efficacy and toxicity, optimal design features of an ADC, translation of PKPD from non-clinical species to patients, and optimal doses and dosing regimens in the clinic. As more clinical data becomes available and our understanding of the PK of ADCs with different types of toxins, linkers and antibody formats improves, we can tackle some of these outstanding questions.
